# Bevacizumab Combined with Platinum–Taxane Chemotherapy as First-Line Treatment for Advanced Ovarian Cancer: Results of the NOGGO Non-Interventional Study (OTILIA) in 824 Patients

**DOI:** 10.3390/cancers13194739

**Published:** 2021-09-22

**Authors:** Jalid Sehouli, Alexander Mustea, Guelten Oskay-Özcelik, Maren Keller, Rolf Richter, Oliver Tomé, Hannah Woopen, Ann-Katrin Sommer-Joos, Jacek P. Grabowski, Robert Armbrust, Pauline Wimberger

**Affiliations:** 1 Department of Gynecology with Center for Oncological Surgery, Corporate Member of Freie Universität Berlin and Humboldt-Universität zu Berlin, Charité—Universitätsmedizin Berlin, 13353 Berlin, Germany; rolf.richter@charite.de (R.R.); hannah.woopen@charite.de (H.W.); jacek.grabowski@charite.de (J.P.G.); robert.armbrust@charite.de (R.A.); 2 Department of Gynecology, Greifswald Medical University, 17489 Greifswald, Germany; mustea@ukbonn.de; 3 Praxisklinik Krebsheilkunde für Frauen, 13597 Berlin, Germany; guelten.oskay@gmx.de; 4 Nord-Ostdeutsche Gesellschaft für Gynäkologische Onkologie (NOGGO) e.V., Schwedenstraße 9, 13359 Berlin, Germany; maren.keller@noggo.de; 5 St Vincentius-Kliniken, 76135 Karlsruhe, Germany; oliver.tome@vincentius-ka.de; 6 Roche Pharma AG, D-79639 Grenzach-Wyhlen, Germany; ann-katrin.sommer-joos@roche.com; 7 Department of Gynecology, Carl Gustav Carus University Dresden, Technische Universität Dresden, 01307 Dresden, Germany; pauline.wimberger@uniklinikum-dresden.de

**Keywords:** bevacizumab, ovarian cancer, anti-angiogenic, platinum–taxane, chemotherapy

## Abstract

**Simple Summary:**

The OTILIA non-interventional study aimed to assess the safety and effectiveness of a standard treatment regimen for advanced ovarian cancer in Germany. All of the women participating in the study received chemotherapy combined with a targeted treatment called bevacizumab. Among the 824 women who received treatment in this study, the median duration of progression-free survival (time alive without their disease returning) was 19.4 months. This is similar to the results in previous randomized phase 3 trials in more restricted populations of women. The safety and effectiveness of treatment seemed to be similar in older (at least 70 years) and younger (less than 70 years) women. Quality of life improved over time.

**Abstract:**

In the single-arm non-interventional OTILIA study, patients with newly diagnosed International Federation of Gynecology and Obstetrics (FIGO) stage IIIB–IV ovarian cancer received bevacizumab (15 mg/kg every 3 weeks for up to 15 months) and standard carboplatin–paclitaxel. The primary aim was to assess safety and progression-free survival (PFS). Subgroup analyses according to age were prespecified. The analysis population included 824 patients (453 aged <70 years, 371 aged ≥70 years). At data cutoff, the median bevacizumab duration was 13.8 months. Grade ≥3 adverse events (AEs), serious AEs, and AEs leading to bevacizumab discontinuation were more common in older than younger patients, whereas treatment-related AEs were less common. Median PFS was 19.4 months, with no clear difference according to age (20.0 vs. 19.3 months in patients <70 vs. ≥70 years, respectively). One-year OS rates were 92% and 90%, respectively. Mean change from baseline in global health status/quality of life showed a clinically meaningful increase over time. In German routine oncology practice, PFS and safety were similar to reported randomized phase 3 bevacizumab trials in more selected populations. There was no notable reduction in effectiveness and tolerability in patients aged ≥70 years; age alone should not preclude use of bevacizumab-containing therapy. ClinicalTrials.gov: NCT01697488.

## 1. Introduction

For many years, the treatment of newly diagnosed ovarian cancer involved cytoreductive surgery and platinum/taxane chemotherapy [1]. In the past decade, incorporation of bevacizumab into front-line chemotherapy regimens has been widely adopted, and is recognized in international guidelines (National Comprehensive Cancer Network, European Society for Medical Oncology-European Society of Gynaecological Oncology) and national guidelines in several countries [2,3,4]. The efficacy and tolerability of front-line bevacizumab combined with carboplatin plus paclitaxel for ovarian cancer were demonstrated in two randomized phase 3 trials: GOG-0218 and ICON7 [5,6]. The results from these trials led to European Medicines Agency (EMA) approval of a regimen of bevacizumab 15 mg/kg every 3 weeks for 15 months in combination with carboplatin and paclitaxel as front-line therapy for stage IIIB–IV ovarian cancer [7]. Subsequently, the U.S. Food and Drug Administration approved the same regimen for stage III or IV ovarian cancer following initial surgical resection [8].

In the Fourth Ovarian Cancer Consensus, bevacizumab-containing regimens were among the options considered to be valid control regimens for phase 3 trials in the front-line setting [9]. The EMA-approved bevacizumab-containing regimen was chosen as the control arm in two recent phase 3 trials: PAOLA-1 evaluating the addition of maintenance olaparib to bevacizumab-containing therapy [10] and IMagyn050 evaluating the addition of atezolizumab to bevacizumab-containing therapy [11]. These trials, together with GOG-0218, provide a rich dataset of more than 1500 women treated with the EMA-approved bevacizumab-containing regimen in the context of randomized phase 3 trials. However, the selected populations eligible for phase 3 clinical trials are not always representative of the scenario in routine clinical practice, where patients tend to be older, less fit, and have more comorbidities [12,13]. To assess the safety and effectiveness of front-line bevacizumab-containing therapy in German clinical practice, we initiated the single-arm non-interventional OTILIA (Ovarian cancer Treatment fIrst-Line wIth Avastin) study (ClinicalTrials.gov identifier: NCT01697488). Previously, we have reported an interim analyses of this study [14,15,16,17,18,19,20]. Here, we present the final results from OTILIA, which, to the best of our knowledge, is the largest analysis of patients with ovarian cancer treated in routine clinical practice.

## 2. Results

### 2.1. Patient Population

A total of 1090 patients were enrolled from 240 sites in Germany between 2 February 2012 and 31 December 2016. Participating sites represented gynecologists, gynecologic oncologists, and oncologists in hospitals and outpatient clinics, as well as office-based oncologists and gynecologists. Of the 1090 enrolled patients, 266 were excluded from the final analysis as they did not meet the inclusion criteria (*n =* 29), received no bevacizumab (*n =* 49), or did not receive bevacizumab according to the approved indication (most commonly no cycle including bevacizumab and both chemotherapy agents together (*n =* 107), bevacizumab frequency not according to the approved indication (*n =* 79), or first bevacizumab dose < 15 mg/kg (*n =* 77)) (more than one reason possible). Thus, the analysis population included 824 patients. In more than two-thirds of these patients (561 patients; 68%), treatment choice was made by a tumor board; the next most frequent decision-makers were office-based oncologists (80 patients; 10%) and gynecologists (76 patients; 9%). The factors most frequently cited as contributing to treatment decision making were guidelines (695 patients; 84%), efficacy of therapy (571 patients, 69%), and study results (404 patients, 49%) (more than one answer possible). These factors were distributed similarly in younger and older patients.

Of the 824 patients in the analysis population, 453 (55%) were aged <70 years and 371 (45%) were aged ≥70 years. All but 45 patients (5%) underwent primary surgery. Compared with patients aged <70 years, those aged ≥70 years tended to have worse Eastern Cooperative Oncology Group performance status (ECOG PS) and were more likely to have pre-existing hypertension (Table 1). Surgical outcome was slightly worse in the older than the younger subgroup.

### 2.2. Treatment Exposure and Documentation

The data cutoff for the final analysis was 27 September 2019. At this date, 453 patients (55%) had discontinued bevacizumab (50% of those aged <70 years and 61% of those aged ≥70 years). The median duration of bevacizumab treatment was 13.8 months (95% confidence interval (CI), 12.7–14.5 months) in the overall population and was slightly longer in younger patients (median 14.6 months, 95% CI, 13.9–15.2 months) than older patients (median 12.5 months, 95% CI, 13.9–15.2 months). The median duration of carboplatin and paclitaxel treatment was 3.5 months (range 0.0–17.7 months for carboplatin, range 0.0–14.1 months for paclitaxel), irrespective of age. Chemotherapy treatment was modified in almost half of the patients (47% of patients had paclitaxel treatment modification, 43% had carboplatin treatment modification), with physician decision most often recorded as the reason for treatment modification. The proportion of patients requiring paclitaxel treatment modification was higher in the older than the younger subgroup (53% vs. 42%, respectively); the difference was less pronounced for carboplatin (45% vs. 41%, respectively). Most of these modifications were treatment delay/interruption rather than a change (increase or reduction) of the dose.

The most common reason for the end of treatment documentation in the overall population was the end of the 15-month documentation period (*n =* 349, 42%), followed by disease progression (*n =* 196, 24%). Reasons for the end of treatment documentation were similar in the younger and older patient subgroups (Table 2).

### 2.3. Safety

Table 3 provides an overview of safety in the overall population and in subgroups according to age. Grade ≥3 adverse events (AEs), serious AEs, and AEs leading to bevacizumab treatment discontinuation were more common in older than younger patients, whereas treatment-related AEs were less common.

**Figure 1:**
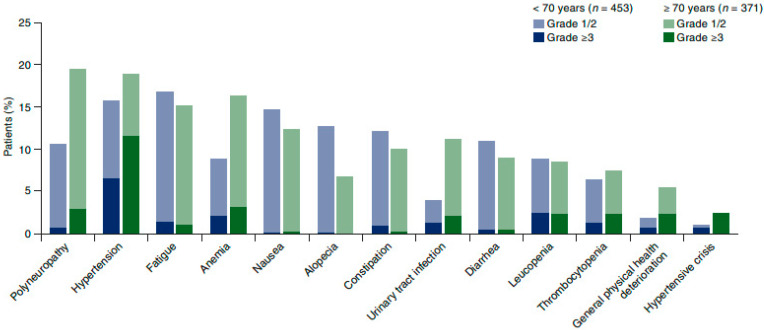
Most common AEs by age. AE, adverse event. Any grade in >10% of patients in either subgroup; grade ≥ 3 in >2% of patients in either subgroup.

In the overall population, the most common AEs were hypertension (17% all grades; 9% grade ≥ 3), fatigue (16% all grades), polyneuropathy (15%), nausea (14%), and anemia (12% all grades, 3% grade ≥ 3). Polyneuropathy, anemia, urinary tract infection, and grade ≥ 3 hypertension were more common in elderly than younger patients; alopecia was less common (Figure 1).

Incidences of other AEs of particular interest in OTILIA were generally low. Proteinuria was reported in 35 patients (4%; considered serious in two patients (0.2%)), large intestine perforation in six patients (0.7%, all considered serious), intestinal perforation in three patients (0.4%, all considered serious), gastric perforation in two patients (0.2%, both considered serious), and arterial embolism in one patient (0.1%, not considered serious).

Among the 30 patients (4%) with fatal AEs (which included unexplained death in six patients (0.7%) and malignant neoplasm progression in four patients (0.5%)), deaths were considered possibly, probably, or definitely related to bevacizumab treatment in five patients (0.6%), comprising urosepsis together with acute kidney injury in one patient aged <70 years, and one case each of cerebrovascular accident in a patient ≥70 years, intestinal perforation in a patient ≥70 years, ileus in a 49-year-old patient, and unexplained death in a 48-year-old patient.

The AE most commonly leading to treatment discontinuation was hypertension (21 patients (2.5%) overall; nine patients (2.0%) aged <70 years vs. 12 (3.2%) aged ≥70 years). The only other AEs leading to treatment discontinuation in ≥1% of patients were proteinuria (10 patients (1.2%) overall; six patients (1.3%) aged <70 years and four (1.1%) aged ≥70 years), polyneuropathy in patients aged ≥70 years (1.6%), and malignant neoplasm progression (recorded as an AE in error) in patients aged ≥70 years (1.1%).

### 2.4. Progression-Free Survival, Overall Response Rate, and Overall Survival

**Figure 2:**
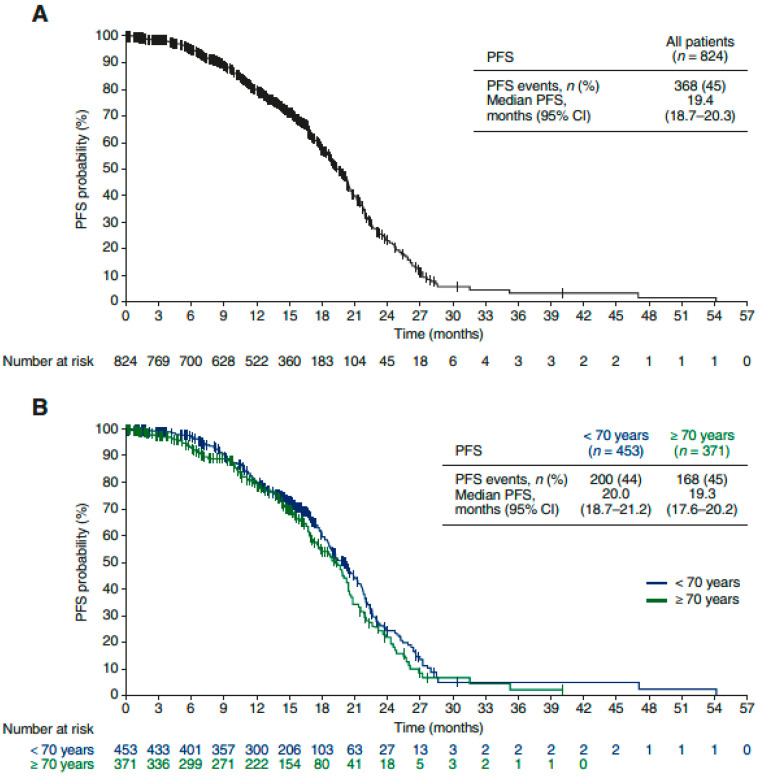
PFS: (**A**) overall population (*n =* 824); (**B**) according to age <70 vs. ≥70 years. CI, confidence interval; PFS, progression-free survival.

At the data cutoff for the final analysis, progression-free survival (PFS) events had been recorded in 368 patients (45%). Median PFS was 19.4 months (95% CI, 18.7–20.3 months) in the overall population (Figure 2A). There was no clear difference in PFS according to age (Figure 2B). Median PFS was 20.0 months (95% CI, 18.7–21.2 months) in patients aged <70 years and 19.3 months (95% CI, 17.6–20.2 months) in patients aged ≥70 years. One-year PFS rates were 80% (95% CI, 76–82%) in the overall population, 80% (76–84%) in the younger subgroup, and 79% (95% CI, 74–83%) in the older subgroup.

In multivariable Cox regression analysis, residual disease at baseline was the only factor shown to be prognostic for PFS (Table S1). Patients with no visible residual disease at baseline had a better PFS outcome than those with residual disease ≥1 cm at baseline (hazard ratio 0.59, 95% CI, 0.45–0.78; *p* < 0.001).

The overall response rate (ORR) in the population of 707 patients assessable for response was 72% (95% CI, 69–75%). Best response was a complete response in 307 patients (43%; 95% CI, 40–47%) and a partial response in 203 patients (29%; 95% CI, 25–32%). The ORR was 77% (95% CI, 73–81%) in 392 assessable patients aged <70 years (including complete response in 50%) and 66% (95% CI, 61–72%) in 315 assessable patients aged ≥70 years (including complete response in 36%).

At the final analysis, deaths had been recorded in 181 patients (22%). Median overall survival (OS) was 24.6 months (95% CI, 23.8–26.3 months), but should be interpreted with caution given the small proportion of patients who had died. The 1-year OS rates were 91% (95% CI, 89–93%) in the overall population, 92% (95% CI, 89–95%) in patients aged <70 years, and 90% (95% CI, 86–93%) in patients aged ≥70 years. Corresponding 2-year OS rates were 53% (46–60%), 60% (50–68%), and 46% (35–55%).

### 2.5. Patient-Reported Outcomes

At baseline, 405 patients (49%) answered at least one item in the quality of life questionnaires. Some additional completed questionnaires could not be included in the analysis because of inaccurate completion of informed consent forms. The participation rate was considerably higher in patients aged <70 years (308 patients; 68%) than those aged ≥70 years (97 patients; 26%). At week 24, 365 patients (44%) completed questionnaires.

Mean change from baseline in global health status/quality of life showed a clinically meaningful increase over time (Figure S1). There were small, but clinically relevant (>10 points [21,22]) improvements over time in role functioning, social functioning, fatigue, nausea and vomiting, and insomnia, with more marked improvements in appetite loss and constipation. European Organisation for Research and Treatment of Cancer Qualify of Life Questionnaire ovarian cancer-specific module (EORTC QLQ-OV28) showed a pronounced increase in peripheral neuropathy and hair loss symptoms within the first 12 weeks of treatment, which lessened or reversed, respectively, over time.

### 2.6. Subsequent Anti-Cancer Therapy

The most common anti-cancer therapies used after bevacizumab-containing front-line therapy were carboplatin (175 patients; 21%), doxorubicin (126 patients; 15%), gemcitabine (108 patients; 13%), and bevacizumab (67 patients; 8%). This pattern was very similar irrespective of age.

## 3. Discussion

We report here the final analysis of routine clinical practice data from patients with advanced ovarian cancer receiving bevacizumab with chemotherapy. This is, to the best of our knowledge, the largest series in primary ovarian cancer and supports the results from phase 3 trials regarding the effectiveness and tolerability of bevacizumab-containing therapy. Median PFS of 19.4 months (95% CI, 18.7–20.3 months) is consistent with the results in the phase 3 GOG-0218 trial using an identical regimen [5]. Median PFS is shorter than the median PFS of 25.5 months (95% CI, 23.7–27.6 months) observed in the international open-label single-arm ROSiA study of bevacizumab (15 mg/kg every 3 weeks in most patients, continued until progression or for up to 24 months) [23], suggesting that a longer bevacizumab treatment duration may improve outcomes. However, recently reported results from the randomized phase 3 AGO-OVAR 17 trial (BOOST; ClinicalTrials.gov Identifier: NCT01462890), which compared the approved regimen of bevacizumab (15 months total duration) with an extended duration (30 months of bevacizumab), undermined this hypothesis [24].

On the other hand, median PFS of 19.4 months in OTILIA is considerably longer than that reported in a similar non-interventional study (OSCAR) in the United Kingdom, in which median PFS was 15.4 months (95% CI, 14.5–16.9 months) [25]. Interestingly, the majority of patients in the OSCAR study received bevacizumab 7.5 mg/kg every 3 weeks for up to 12 months, consistent with the regimen evaluated in ICON7 and frequently used in U.K. practice. Another important difference is the surgical approach; while 95% of patients in the German OTILIA study had primary cytoreductive surgery, in the U.K. OSCAR study, only 21% had primary cytoreductive surgery, 36% received interval surgery, and a remarkable 43% received no surgery at all. The significant prognostic effect of cytoreduction to no visible residual disease was again highlighted in our study, thus this is likely to be an important factor contributing to the marked differences in outcome between our study and the OSCAR study. The results from the French ENCOURAGE non-interventional study in routine practice have not yet been published in full, but median PFS was reported to be 17.4 months (95% CI, 16.4–19.1 months) in 468 patients treated with front-line bevacizumab-containing therapy (15 mg/kg every 3 weeks in 80% of patients) [26]. The Italian single-arm MITO-16A/MaNGO-OV2A study reported median PFS of 20.8 months (95% CI 19.1–22.0 months) in 398 patients [27]. In the PAOLA-1 randomized phase 3 trial, median PFS in the control arm (identical to the regimen used in OTILIA) was 16.6 months [10] in a study population undergoing upfront surgery in 51% and interval surgery in 41% of patients (no surgery in 8%). The addition of maintenance olaparib increased median PFS to 22.1 months. Finally, in the IMagyn050 trial, median PFS in the control arm was 18.4 months (95% CI, 17.2–19.8 months) vs. 19.5 months (95% CI, 18.1–20.8 months) in the atezolizumab arm [11].

Similar to subgroup analyses from the ROSiA and OSCAR studies [25,28], age had no clear impact on clinical outcomes (PFS or OS) among patients receiving front-line bevacizumab-containing therapy. Although some selection bias is likely even in a routine clinical practice study, this finding is encouraging for clinicians faced with treatment decisions for patients aged ≥70 years.

AEs were consistent with the well-defined safety profile of bevacizumab in ovarian cancer and bevacizumab was well tolerated in patients aged ≥70 years as well as in younger patients. The most common AE was hypertension, but this rarely led to treatment discontinuation. Hypertension was more common in older than younger patients, as expected given the higher prevalence of pre-existing hypertension in the older subgroup and the known association between age and hypertension, irrespective of treatment. This finding is consistent with observations in the ROSiA and OSCAR studies in the front-line setting [25,28]. The tolerability of bevacizumab-containing therapy in older patients is also consistent with Amadio et al.’s conclusions from a case-control study in patients receiving bevacizumab in either the front-line or the recurrent setting [29]. Grade ≥3 AEs were no more frequent in 72 patients aged ≥65 years than in 211 aged <65 years. However, creatinine serum concentration, estimated glomerular filtration rate, and presence of ≥3 comorbidities were independently associated with increased toxicity, and may be more relevant in patient selection for bevacizumab-containing therapy than chronological age.

Patient-reported outcomes (PROs) from OTILIA support the tolerability of front-line bevacizumab-containing therapy in routine practice, showing no deterioration and even a slight improvement over time in some scales. However, PRO conclusions are limited by the low participation rate, particularly in older patients.

Another limitation of the analyses is the relatively restricted follow-up period. In accordance with the legal requirements for non-interventional studies in Germany, patients cannot be followed for >27 months. Consequently, PFS results were reported after events in less than half of the population and OS results are very immature. The OTILIA study was initiated in 2012, pre-dating the 2014 revision of International Federation of Gynecology and Obstetrics (FIGO) staging into high-grade serous and low-grade serous tumors [30]. Consequently, details of histology and tumor grade at diagnosis are limited. There was also no information on molecular markers, such as *BRCA* status or homologous recombination deficiency status, which are known to have prognostic value and affect treatment decisions in today’s clinical practice. The addition of maintenance olaparib to bevacizumab-containing front-line therapy significantly improves PFS, particularly in patients with *BRCA*-mutated tumors. Our results confirm the importance of this effective bevacizumab-containing regimen as a foundation for polyADP ribose polymerase (PARP) inhibitor-containing strategies and future clinical trials in the front-line setting.

## 4. Patients and Methods

This single-arm open-label multicenter non-interventional study aimed to assess the effectiveness, safety, tolerability, and impact on PROs of front-line bevacizumab in combination with carboplatin plus paclitaxel chemotherapy in routine oncology practice in Germany. Eligible patients had newly diagnosed FIGO stage IIIB–IV ovarian cancer and received front-line bevacizumab, carboplatin, and paclitaxel combination therapy according to the EMA label [7]. Patients with any contraindication to bevacizumab treatment according to the approved indication were ineligible.

All patients provided written informed consent. Participation had no influence on the therapeutic and diagnostic management of patients in this non-interventional study. Ethics advice was obtained in accordance with the laws and regulations applicable in Germany and according to the World Medical Association Declaration of Helsinki.

The primary aim was to assess safety and PFS (defined as the interval between the first dose of bevacizumab and disease progression or death from any cause). Secondary objectives were to evaluate ORR and OS; to determine the frequencies of AEs, serious AEs, and adverse drug reactions with a special focus on arterial hypertension, arterial thromboembolism, gastrointestinal perforations, and proteinuria; as well as to assess factors influencing treatment choice, treatment exposure (treatment duration, incidence, and reasons for treatment modification and discontinuation), and quality of life. In a protocol amendment implemented in July 2014, the trial was expanded to focus on patients aged ≥70 years. The aim was to investigate whether the effectiveness and safety of bevacizumab-containing therapy in routine practice replicated observations in the randomized clinical trials.

Study data were recorded by the treating physician in an electronic case report form, and included demographic data, medical history, prior therapy, comorbidities, treatment regimen and exposure, reasons for treatment selection, concomitant medication, tumor evaluation, details of AEs, reasons for treatment and study discontinuation, and PROs. AEs were recorded at every cycle and graded according to National Cancer Institute Common Terminology Criteria for Adverse Events (version 4.0). Tumor assessment was performed according to the investigator’s judgement and local practice. PROs were assessed with the EORTC QLQ core module (QLQ-C30) and QLQ-OV28 using paper-based questionnaires. Missing items were imputed by the average of the answered items, if answers for at least half of the items of a score were available. If more than half of the items of a score were missing, the score was set to missing. During the first 15 months, information was documented at each treatment cycle. Subsequently, patients were followed for 12 months after disease progression, discontinuation of bevacizumab, or completion of 15 months of bevacizumab therapy (i.e., a maximum of 27 months of documentation for each patient). During the 12-month follow-up period, documentation was less intense (only every 6 months and collecting only limited information, such as patient status, tumor evaluation, and further anti-cancer therapy).

Statistical analysis of this non-interventional study was exploratory, primarily using descriptive statistical methods. A target sample size of 1190 patients was calculated based on an assumed median PFS in the overall population of 19 months (based on the results from ICON7 and GOG-0218) and an observation period of 27 months for each patient, together with the objective of determining PFS in the subgroup of patients aged ≥70 years. This sample size provided 95% probability of observing AEs with an incidence of 0.3%.

There was no adjustment for multiplicity. All time-related endpoints (PFS, OS, treatment duration) were analyzed using Kaplan–Meier methodology, with medians presented together with associated 95% CIs. PFS rates at 6, 12, and 18 months were calculated. A multivariate Cox regression analysis was performed to assess the effect of selected covariates on PFS, including the baseline covariates age, ECOG PS, body mass index, residual disease, FIGO stage, ascites, and tumor grade. The analysis population for effectiveness and safety included all patients who received at least one dose of bevacizumab according to the approved indication. Analyses of PROs were based on the subset of these patients who consented to participate in the quality of life assessment. Subgroup analyses were performed according to age (<70 vs. ≥70 years).

## 5. Conclusions

In routine oncology practice in Germany, we observed PFS and a safety profile similar to those demonstrated in randomized phase 3 trials in more restricted, highly selected populations, confirming the value of this treatment strategy for women presenting with newly diagnosed ovarian cancer in the real-world setting. There was no notable reduction in effectiveness and tolerability in patients aged ≥70 years; therefore, age alone should not preclude use of bevacizumab-containing therapy.

## Figures and Tables

**Table 1:** Patient and disease characteristics at baseline.

Characteristic		All (*n* = 824)	Age < 70 Years (*n* = 453)	Age ≥ 70 Years (*n* = 371)
Median age, years (range)		68 (26–83)	58 (26–70 *)	75 (70–83)
Median BMI, kg/m^2^ (range)		24.2	23.9	24.6
		(15.6–43.5)	(16.2–43.5)	(15.6–42.3)
ECOG PS, *n* (%)	0	297 (36)	199 (44)	98 (26)
	1	389 (47)	194 (43)	195 (53)
	2	77 (9)	36 (8)	41 (11)
	3	15 (2)	6 (1)	9 (2)
	Missing	46 (6)	18 (4)	28 (8)
Hypertension, *n* (%)		339 (41)	132 (29)	207 (56)
Current treatment for hypertension, *n* (%)		312 (38)	117 (26)	195 (53)
FIGO stage, *n* (%)	IIIB	116 (14)	65 (14)	51 (14)
	IIIC	472 (57)	265 (58)	207 (56)
	IV	236 (29)	123 (27)	113 (30)
Postoperative residual disease, *n* (%)	No macroscopic	229 (28)	139 (31)	90 (24)
	0–≤1 cm	175 (21)	106 (23)	69 (19)
	>1 cm	192 (23)	99 (22)	93 (25)
	Unknown	183 (22)	96 (21)	87 (23)
	Missing	45 (5)	13 (3)	32 (9)
Primary cancer, *n* (%)	Epithelial ovary	662 (80)	367 (81)	295 (80)
	Fallopian tube	58 (7)	27 (6)	31 (8)
	Primary peritoneal	104 (13)	59 (13)	45 (12)
Histological subtype, *n* (%)	Serous	606 (74)	333 (74)	273 (74)
	Endometrioid	22 (3)	14 (3)	8 (2)
	Mucinous	19 (2)	13 (3)	6 (2)
	Clear cell	13 (2)	9 (2)	4 (1)
	Undifferentiated	24 (3)	13 (3)	11 (3)
	Other	95 (12)	58 (13)	37 (10)
	Missing	45 (5)	13 (3)	32 (9)
Tumor grade, *n* (%)	Low	22 (3)	13 (3)	9 (2)
	Intermediate	153 (19)	99 (22)	54 (15)
	High	577 (70)	314 (69)	263 (71)
	Not assessable	72 (9)	27 (6)	45 (12)

Abbreviations: BMI, body mass index; ECOG PS, Eastern Cooperative Oncology Group performance status; FIGO, International Federation of Gynecology and Obstetrics. * Two patients aged <70 years at enrolment were aged ≥70 years at the start of therapy.

**Table 2:** Treatment exposure and documentation.

Reason for End of Treatment Documentation, *n* (%) *	All (*n* = 824)	Age < 70 Years (*n* = 453)	Age ≥ 70 Years (*n* = 371)
End of documentation after 15 months	349 (42)	213 (47)	136 (37)
Disease progression	196 (24)	105 (23)	91 (25)
Patient request (no toxicity)	53 (6)	24 (5)	29 (8)
AE ^†^	44 (5)	30 (7)	14 (4)
AE related to treatment ^†^	40 (5)	11 (2)	29 (8)
AE not related to treatment ^†^	25 (3)	8 (2)	17 (5)
No end of treatment documentation	18 (2)	6 (1)	12 (3)
Death	16 (2)	5 (1)	11 (3)
Lost to follow-up	15 (2)	10 (2)	5 (1)
Tumor remission	13 (2)	7 (2)	6 (2)
Patient request	7 (1)	3 (1)	4 (1)
Other	48 (6)	31 (7)	17 (5)

Abbreviation: AE, adverse event. * Multiple answers possible. ^†^ Before 1 October 2013, it was not possible to specify whether or not an AE was related to treatment; from 1 October 2013, attribution to treatment was possible.

**Table 3:** Overview of safety.

AE, *n* (%)	All (*n* = 824)	Age < 70 Years (*n* = 453)	Age ≥ 70 Years (*n* = 371)
All grades	616 (75)	328 (72)	288 (78)
Grade ≥3	317 (38)	150 (33)	167 (45)
Grade 5	30 (4)	12 (3)	18 (5)
Treatment-related AEs	330 (40)	192 (42)	138 (37)
Serious AEs	222 (27)	100 (22)	122 (33)
Treatment-related serious AEs	72 (9)	37 (8)	35 (9)
AEs leading to bevacizumab discontinuation	145 (18)	66 (15)	79 (21)

Abbreviation: AE, adverse event.

## Data Availability

Qualified researchers may request access to individual patient-level data through the clinical study data request platform (https://vivli.org/). Further details on Roche’s criteria for eligible studies are available here (https://vivli.org/members/ourmembers/). For further details on Roche’s Global Policy on the Sharing of Clinical Information and how to request access to related clinical study documents, see here (https://www.roche.com/research_and_development/who_we_are_how_we_work/clinical_trials/our_commitment_to_data_sharing.htm).

## Supplementary Materials

The following are available online at https://www.mdpi.com/article/10.3390/cancers13194739/s1, Figure S1: Mean change from baseline in European Organisation for Research and Treatment of Cancer Quality of Life Questionnaire Core Module Global Health Status/Quality of Life, Table S1: Multivariable cox regression model of PFS.
