# Supplementary material for: Bevacizumab Combined with Platinum–Taxane Chemotherapy as First-Line Treatment for Advanced Ovarian Cancer: Results of the NOGGO Non-Interventional Study (OTILIA) in 824 Patients
Source: Cancers (Basel). 2021 Sep 22;13(19):4739. doi: 10.3390/cancers13194739 (PMC8507543; doi:10.3390/cancers13194739)
Supplement: Supplementary file 1 [file cancers-13-04739-s001.zip › cancers-1365850-supplementary.pdf]

Supplementary Materials: Bevacizumab Combined with Platinum–Taxane Chemotherapy as First-Line Treatment for Advanced Ovarian Cancer: Results of the NOGGO Non-Interventional Study (OTILIA) in 824 Patients

Jalid Sehouli, Alexander Mustea, Guelten Oskay-Özcelik, Maren Keller, Rolf Richter, Oliver Tomé, Hannah Woopen, Ann-Katrin Sommer-Joos, Jacek P. Grabowski, Robert Armbrust and Pauline Wimberger, on behalf of the North-Eastern German Society of Gynaecological Oncology (NOGGO)

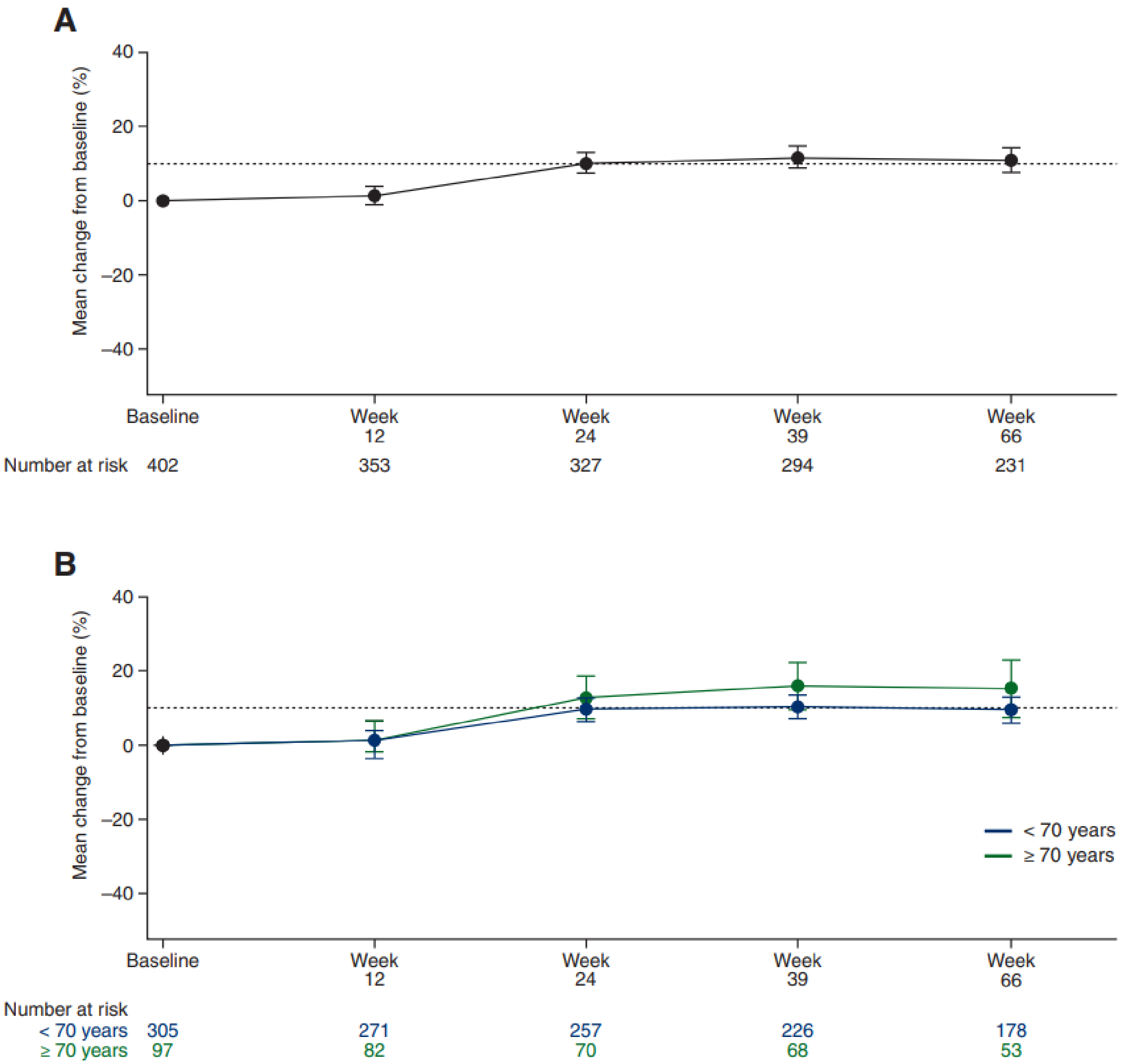

Figure S1. Mean change from baseline in European Organisation for Research and Treatment of Cancer Quality of Life Questionnaire Core Module Global Health Status/Quality of Life: (A) overall population (n = 402); (B) according to age < 70 vs ≥ 70 years. Dotted line represents increase of 10 percentage points from baseline (clinically relevant increase).

Table S1. Multivariable cox regression model of PFS.

Variable		PFS Hazard Ratio (95% CI)
**ECOG PS**	>1 vs 0/1	1.05 (0.75–1.49)
	Unknown vs 0/1	1.10 (0.69–1.75)
BMI, kg/m^2	≤20 vs >20–25	1.06 (0.76–1.49)
	>25–30 vs >20–25	0.94 (0.72–1.21)
	>30 vs >20–25	1.11 (0.82–1.51)
	Unknown vs >20–25	0.65 (0.33–1.25)
Age	≥70 vs <70 years	1.16 (0.94–1.44)
Residual disease at baseline	No visible residuum vs ≥1 cm	0.59 (0.45–0.78)
	Unknown vs ≥1 cm	0.80 (0.60–1.07)
FIGO stage	IIIB vs IIIC/IV	0.79 (0.56–1.11)
Ascites at baseline, mL	0 vs <0–500	1.53 (0.53–4.44)
	>500 vs >0–500	1.11 (0.74–1.67)
	Unknown vs >0–500	0.97 (0.68–1.36)
Grade	Well differentiated vs moderately/poorly differentiated	0.74 (0.32–1.68)
	Undifferentiated/unknown vs moderately/poorly differentiated	0.88 (0.61–1.25)
Prior surgery	No vs yes	1.25 (0.77–2.02)

Abbreviations: BMI, body mass index; CI, confidence interval; ECOG PS, Eastern Cooperative Oncology Group performance status; FIGO, International Federation of Gynecology and Obstetrics; PFS, progression-free survival.
